# Intestinal *Candida parapsilosis* isolates from Rett syndrome subjects bear potential virulent traits and capacity to persist within the host

**DOI:** 10.1186/s12876-018-0785-z

**Published:** 2018-05-02

**Authors:** Francesco Strati, Antonio Calabrò, Claudio Donati, Claudio De Felice, Joussef Hayek, Olivier Jousson, Silvia Leoncini, Daniela Renzi, Lisa Rizzetto, Carlotta De Filippo, Duccio Cavalieri

**Affiliations:** 1 Computational Biology Research Unit, Research and Innovation Centre, Fondazione Edmund Mach, Via E. Mach 1, 38010, San Michele all’Adige, Italy; 2 Centre for Integrative Biology, University of Trento, Via Sommarive 9, 38123 Trento, Italy; 3 Department of Experimental and Clinical Biomedical Sciences, Gastroenterology Unit, University of Florence, Viale Morgagni 40, 50139 Florence, Italy; 4 Neonatal Intensive Care Unit, University Hospital AOUS, Viale Bracci 16, 53100 Siena, Italy; 5 Child Neuropsychiatry Unit, University Hospital AOUS, Viale Bracci 16, 53100 Siena, Italy; 6 Nutrition and Nutrigenomics Unit, Research and Innovation Centre, Fondazione Edmund Mach, Via E. Mach 1, 38010 San Michele all’Adige, Italy; 7 Institute of Agriculture Biology and Biotechnology (IBBA), National Research Council (CNR), Via Moruzzi 1, 56124 Pisa, Italy; 8 Department of Biology, University of Florence, Via Madonna del Piano 6, 50019 Sesto Fiorentino, Florence Italy; 9 Present address: T Cell Development Lab, Institute for Research in Biomedicine, Università della Svizzera Italiana, Via Vincenzo Vela 6, CH-6500 Bellinzona, Switzerland

**Keywords:** Rett syndrome, *Candida parapsilosis*, Dysbiosis

## Abstract

**Background:**

Rett syndrome (RTT) is a neurological disorder mainly caused by mutations in *MeCP2* gene. It has been shown that MeCP2 impairments can lead to cytokine dysregulation due to MeCP2 regulatory role in T-helper and T-reg mediated responses, thus contributing to the pro-inflammatory status associated with RTT. Furthermore, RTT subjects suffer from an intestinal dysbiosis characterized by an abnormal expansion of the *Candida* population, a known factor responsible for the hyper-activation of pro-inflammatory immune responses. Therefore, we asked whether the intestinal fungal population of RTT subjects might contribute the sub-inflammatory status triggered by MeCP2 deficiency.

**Methods:**

We evaluated the cultivable gut mycobiota from a cohort of 50 RTT patients and 29 healthy controls characterizing the faecal fungal isolates for their virulence-related traits, antifungal resistance and immune reactivity in order to elucidate the role of fungi in RTT’s intestinal dysbiosis and gastrointestinal physiology.

**Results:**

*Candida parapsilosis*, the most abundant yeast species in RTT subjects, showed distinct genotypic profiles if compared to healthy controls’ isolates as measured by hierarchical clustering analysis from RAPD genotyping. Their phenotypical analysis revealed that RTT’s isolates produced more biofilm and were significantly more resistant to azole antifungals compared to the isolates from the healthy controls. In addition, the high levels of IL-1β and IL-10 produced by peripheral blood mononuclear cells and the mixed Th1/Th17 cells population induced by RTT *C. parapsilosis* isolates suggest the capacity of these intestinal fungi to persist within the host, being potentially involved in chronic, pro-inflammatory responses.

**Conclusions:**

Here we demonstrated that intestinal *C. parapsilosis* isolates from RTT subjects hold phenotypic traits that might favour the previously observed low-grade intestinal inflammatory status associated with RTT. Therefore, the presence of putative virulent, pro-inflammatory *C. parapsilosis* strains in RTT could represent an additional factor in RTT’s gastrointestinal pathophysiology, whose mechanisms are not yet clearly understood.

**Electronic supplementary material:**

The online version of this article (10.1186/s12876-018-0785-z) contains supplementary material, which is available to authorized users.

## Background

The gut mycobiota, together with the bacterial microbiota, exerts key roles in maintaining the intestinal microbial community structure, metabolic functions and has strong immunomodulatory properties, being a main actor in host physiopathology [1]. The host response to fungi is mediated at first by the innate immunity through recognition of the fungal pathogen-associated molecular patterns (PAMPs) by the host cells’ pattern recognition receptors (PRRs). The C-type lectin receptors (CLRs; e.g. dectin 1 and dectin 2, also known as CLEC7A and CLEC6A respectively) are fundamental for fungal recognition and for the development of innate and adaptive immune responses, especially T-helper (Th) 1 and Th17 responses [2]. Th1 cells, through the production of IFNγ and TNFα, activates and recruits phagocytes (macrophages, neutrophils) at potential sites of infection [2], while Th17 cells are necessary for protection against fungal infections [3, 4]. In fact, IL-17 ability to mobilize neutrophils and to induce the production of antimicrobial peptides contributes to an efficient control of fungi at different body sites [2]. However, well balanced pro-inflammatory and tolerogenic responses are a prerequisite to avoid potential harmful inflammatory responses triggered by gastrointestinal fungi. The shift between pro-inflammatory and tolerogenic dendritic cells (DCs) responses are mediated by the kynurenine pathway of tryptophan catabolism, in which the expression of indoleamine 2,3-dioxygenase (IDO1) has a key role on plasticity of DCs activities in balancing between CD4^+^ effector Th cells and regulatory T (T-reg) cells [2, 5]. IDO1 is widely recognized as a regulator of immune homeostasis and suppressor of inflammation by inducing IL-10 through the production of immune-active kynurenines that activate the aryl-hydrocarbon receptor (AHR) in lymphoid tissues [6], thus inducing the transcription of FOXP3 and promoting immune tolerance via T-reg cells [7, 8]. Nevertheless, the mechanisms by which commensal fungi choose to shift their phenotype towards infection are not well understood, even if the disruption of the microbial community structure resulting in intestinal dysbiosis has been proposed to be one of the reasons [9]. Indeed, alterations of the gut microbiota can lead to inflammation involving hyper activation of Th1 and Th17 immune responses [10]. Altered immunological response to fungi can in turn contribute to systemic inflammatory responses. Remarkably, fungal infections shift IDO1’s activity [11, 12], reducing the levels of kynurenine and thus promoting inflammation [13]. Furthermore, alterations of the levels of kynurenine, a neuroprotective agent, have been implicated in several pathologies, including autism spectrum disorders [14].

Rett syndrome (RTT) is a neurological disorder that almost exclusively affects females with an incidence of 1:10,000 live births [15] due to a loss-of-function mutations of the X-linked methyl-CpG binding protein 2 (*MeCP2*) gene in approximately 90% of classic RTT cases [16]. RTT subjects develop normally up to 18 months of age after which they undergo a period of neurological regression [15]. RTT affects several organs and systems among which the autonomic nervous system [15], the gastrointestinal tract [17] and the immune system [18] making it eligible as a multisystemic disease [15]. It has been shown that MeCP2 deficiency is able to lead to cytokine dysregulation [18, 19], to influence the expression of FOXP3 [20], an important transcription factor involved in the generation of T-reg cells, and to determine the significant increase of secreted IL-17A [20]. Since the Th17/T-reg balance is implicated in the development of autoimmune/inflammatory disorders it is possible to hypothesize the presence of an autoimmune component in RTT [21]. To this regard, intestinal dysbiosis may cause chronic intestinal inflammation and autoimmunity as occurring in Inflammatory Bowel Diseases (IBDs) [22]. Previous studies indicated the presence of a subclinical inflammatory status in subjects affected by RTT [23] remarked by cytokine dysregulation in both Th1 and Th17 responses [19, 20, 24, 25] and an intestinal dysbiosis characterized by high relative abundance of the genus *Candida* [26]. Therefore, we asked whether the intestinal fungal population of RTT subjects might be involved in the sub-inflammatory status triggered by MeCP2 deficiency. Here we studied the cultivable gut mycobiota of RTT subjects characterizing the isolated fungi for their virulence-related traits and antifungal resistance. Moreover, we characterized the genetic diversity of *C. albicans* and *C. parapsilosis* isolates and their ability to induce innate and adaptive immunological responses in human PBMCs in order to elucidate the role of fungi in RTT gastrointestinal pathophysiology.

## Methods

### Isolation and identification of cultivable fungal species from faeces

The participants’ data related to the 50 RTT patients and 29 Healthy Controls (HC) included in this study are available in [26]. Stool samples from enrolled subjects [26] were collected, aliquoted as it is and stored at − 80 °C until analysis. Samples were homogenized in sterile Ringer’s solution and plated on solid YPD medium (1% Yeast extract, 2% Bacto-peptone, 2% D-glucose, 2% agar) supplemented with 25 U/ml of penicillin, 25 μg/ml of streptomycin (Sigma-Aldrich) and incubated aerobically at 27 °C for 3–5 days. All fungal isolates grown on the selective medium were further isolated to obtain single-cell pure colonies. Genomic DNA was extracted from pure cultures of the isolated colonies as previously described [27]. Fungal isolates were identified by amplification and sequencing of the ribosomal Internal Transcribed Spacer (ITS) region, using ITS1 (5’-GTTTCCGTAGGTGAACTTGC-3′) and ITS4 (5’-TCCTCCGCTTATTGATATGC-3′) primers [28]. ITS1–4 sequences were then classified by using the BLAST algorithm in the NCBI database (minimum 97% sequence similarity and 95% coverage with a described species).

### Invasive growth

The ability of fungal strains to penetrate YPD solid medium was tested as previously described [29]. M28-4D and BY4742 *S. cerevisiae* strains, known to be invasive and non-invasive respectively, were used as controls. The strain invasiveness was assigned with scores from 3 (highly invasive) to 0 (non-invasive).

### Hyphal formation

Fungal cells (~ 10^5^ cells/ml) were grown for 7 days in liquid YPD and YNB media (0.67% Yeast Nitrogen Base w/o aminoacids and (NH_4_)_2_SO_4_ (Sigma-Aldrich)_,_ 2% glucose), both at 27 °C and 37 °C in order to evaluate hyphae or pseudohyphae formation. Formation of hyphae was inspected by optical microscope observation with a Leica DM1000 led instrument (magnification 40× and 100×) [30].

### Biofilm formation

Biofilm formation was quantified according to a previous published protocol [31]. Briefly, fungal cells (~ 10^5^ cells/ml) were grown in liquid YPD at 37 °C for 48 h in flat-bottom 96-well plates. After the incubation period, cell suspensions were aspirated and each well with the adhered fungal cells was washed three times with deionized H_2_O and one time with PBS 1X. Biofilm-coated wells were then incubated with 0.01% of crystal violet (Sigma) for 30 min and washed as above. Finally, each well of the dried 96-well plate was incubated with 100 μl of 100% EtOH for 10 min and biofilm formation was quantified by optical density measurement at 570 nm with a microplate reader (Synergy2, BioTek, USA).

#### Antifungal susceptibility testing

All fungal isolates were tested for susceptibility to fluconazole, itraconazole and 5-flucytosine (Sigma-Aldrich) by Minimum Inibitory Concentration (MIC) assays according to the European Committee on Antimicrobial Susceptibility Testing (EUCAST) recommendations [32, 33]. EUCAST clinical breakpoints (CBPs) were used to evaluate the antifungal resistance. Although CBPs have not been established for non-*Candida* yeasts and the non-*Aspergillus* moulds, they have been used as a proxy for the evaluation of antifungals susceptibility in such isolates.

### RAPD genotyping and clustering analysis

*Candida albicans* and *C. parapsilosis* isolates were genotyped by Random Amplification of Polymorphic DNA (RAPD) using the primer Oligo 2 (5’-TCACGATGCA-3′) as described previously [34]. Amplifications were performed according to the following protocol: 5 min at 94 °C, 40 cycles of 30s at 94 °C, 30s at 36 °C and 2 min at 72 °C, followed by a final extension of 10 min at 72 °C. The PCR reaction mix contained 1X PCR buffer 2 mM MgCl_2_, 200 μM of dNTPs, 0.4 μM of the primer, 2.5 U of Taq Polymerase and 10 ng of gDNA as template. PCR amplicons were separated using a 2% agarose gel in 1× TAE buffer at 90 V for 2 h and visualized with 0.5 μg/ml ethidium bromide staining. The presence or absence of an amplicon at any position of the gel was used for the construction of a binary matrix for the calculation of samples’ distance similarity according to the Jaccard index [35] by mean of the “vegdist” function within the *vegan* R package. The samples have been then clustered hierarchically according to the UPGMA method by using the “hclust” function within the *stats* R package.

### Isolation and stimulation of PBMCs

Human peripheral blood mononuclear cells (PBMCs) were isolated by Ficoll-Hypaque density gradient centrifugation (Biochrom, Berlin, Germany) from buffy coats provided by the Transfusion Unit of Ospedale Santa Chiara in Trento, Italy. The experimental plan was approved by the local hospital ethical committee, and informed consent was obtained from all the healthy donors (protocol No: 54896583). *Candida* isolates were cultured in YPD medium for 18 h at 37 °C. Fungal cells were harvested by centrifugation, washed twice with PBS, heat-killed for 3 h at 65 °C and resuspended in culture medium (RPMI1640; Sigma Aldrich). For stimulation experiments, 5 × 10^5^ PBMCs in RPMI1640 were incubated with 5 × 10^6^ heat-killed *C. albicans*, *C. parapsilosis* or RPMI1640 medium alone (negative control) [36]. After the incubation periods (24 h for IL-1β, IL-6, TNF-α, IL-10 production and 120 h for IL-17A, INFγ, IL-22, IL-10 production) cell suspensions were centrifuged and supernatants were collected and stored at − 20 °C until assayed. Each experiment was performed in triplicate.

### Cytokine assays

Cytokine detection i.e. IL-17A, INFγ, IL-22, IL-1β, IL-6, TNF-α, IL-10 production, were assayed using the MAP human cytokine/chemokine kit (Merck Millipore) according to the manufacturer’s instructions (MagPix technology).

### Flow cytometry

PBMCs were collected after stimulation with *Candida* isolates in a ratio of 10:1 (stimuli:cells) and washed with PBS. Intracellular staining for IDO1 (after 24 h of stimulation), T-bet and RORγt (after 5 day of stimulation) were performed using the fixation/permeabilization buffer kit (Life Technologies) following the manufacturing recommendations. Cells were then stained with adequate concentrations of labelled antibodies diluted in PBS with 10% heat-inactivated foetal bovine serum (FBS) for 20 min at room temperature, A minimum of ten thousand events for each sample were acquired using a Guava easyCyte 8 T flow cytometer (Merck Millipore) and analysed using the inCyte software (Merck Millipore). Cells were gated first based on forward and side scatter to exclude dead cells and cell debris. The area of positivity was determined by using an isotype-matched control MAb. Antibodies used: Fluorescein isothiocyanate (FITC)-IDO1 (BD Biosciences Pharmingen, Prodotti Gianni, Italy), FITC-Tbet (Millipore), allophycocyanin (APC)-RORγt (BD Biosciences Pharmingen, Prodotti Gianni, Italy).

### Statistical analysis

Wilcoxon rank-sum tests and Spearman’s correlations were performed using the R software [37] through the *stats* R package (version 3.1.2) and the *psych* R package, respectively. Permutational MANOVA (PERMANOVA) test was performed using the adonis()function of the *vegan* R package with 999 permutations. All *p-*values have been corrected for multiple hypothesis controlling the false discovery rate (FDR) [38].

## Results

### RTT gut mycobiota shows a reduction of *C. albicans* and an increase of *C. parapsilosis* populations

**Fig. 1:**
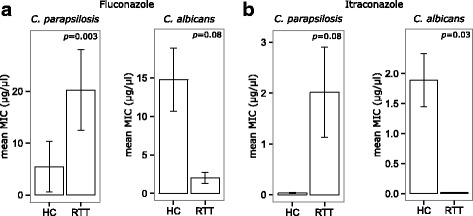
**a**) Fluconazole and **b**) itraconazole resistance as measured by MIC values in *C. albicans* and *C. parapsilosis* isolates from HC and RTT subjects. MIC values are reported as means ± standard errors. Exact *p-values* are reported and considered significant if < 0.05

We identified 122 fungal isolates belonging to different species (Additional file 1: Table S1). Twenty-four of such isolates were obtained from stool samples of RTT subjects (Additional file 1: Table S1). We discovered a significant reduction of fungal species richness in RTT subjects compared to HC (*p* = 3.9e-05, Wilcoxon rank-sum test) in agreement with the results obtained using a metagenomic approach from the same study cohort [26]. *Candida* was the most abundant genus present in both RTT subjects (91.7%) and HC (71.4%) with *C. albicans* and *C. parapsilosis* as the two most abundant species in both RTT subjects and HC. Interestingly, we observed an inversion in the relative abundances of *C. albicans* and *C. parapsilosis* between RTT subjects and HC. While in RTT subjects 4 out of 24 fungal isolates belonged to *C. albicans* (16.7%) and 14 out of 24 belonged to *C. parapsilosis* (58.3%), in the HC 49 out of 98 fungal isolates belonged to *C. albicans* (50%) and 15 out of 98 belonged to *C. parapsilosis* (15.3%) (Additional file 2: Figure S1). We then characterized the fungal isolates for putative virulence-associated traits and resistance to antifungals (Additional file 1 Table S1). We found that 50% and 63.8% of fungal isolates from RTT subjects and HC, respectively, were able to form hyphae or pseudohyphae (Additional file 1: Table S1). In addition, we observed that the morphotype switch to hyphae or pseudohyphae was related to the isolates’ invasiveness, with hyphae- and pseudohyphae-forming isolates being the most invasive (Additional file 3: Figure S2A). We also observed that RTT isolates produced more biofilm (*p* = 1.3e-05, Wilcoxon rank-sum test; Additional file 3: Figure S2B) and were significantly more resistant to fluconazole compared to HC isolates (45.8% of RTT isolates were resistant vs 18.1% of HC isolates; *p* = 5.1e-06, Wilcoxon rank-sum test). As previously observed, we found the co-occurrence of azole cross-resistance between fluconazole and itraconazole (Spearman’s correlation *r* = 0.57; *p* = 2.2e-10) [39]. Almost the totality of the isolates (96.7%) were susceptible to 5-flucytosine, with MIC≤0.125 μg/ml (Additional file 1: Table S1). *Candida parapsilosis* isolates from HC were susceptible to fluconazole (MIC_90_ = 2 μg/ml; *R* = 7.1%) and itraconazole (MIC_90_ = 0.0156 μg/ml; *R* = 0%) while *C. parapsilosis* isolates from RTT subjects exhibited a high resistance to these antifungals (fluconazole, MIC_90_ > 64 μg/ml, *R* = 35.7%, *p* = 0.003, Wilcoxon rank-sum test, Figure 1A; itraconazole, MIC_90_ > 8 μg/ml, R = 35.7%; Table 1). On the contrary, *C. albicans* isolates from HC were resistant to fluconazole (MIC_90_ > 64 μg/ml, *R* = 24.4%) and itraconazole (MIC_90_ > 8 μg/ml, *R* = 63.4%; *p* = 0.03, Wilcoxon rank-sum test; Fig. 1B) while only one RTT *C. albicans* isolate was resistant to fluconazole (Table 1). Noteworthy, all other *Candida* species isolated from RTT subjects (i.e. *C. glabrata, C. pararugosa* and *C. tropicalis*) were resistant to fluconazole (MIC90 > 64 μg/ml; *R* = 100%) and itraconazole (MIC90 = 8 μg/ml; R = 100%) while *Candida* spp. isolated from HC (i.e. *C. deformans, C. intermedia* and *C. lusitaniae*) were completely susceptible to these azoles (Table 1). Taken together these results suggest that RTT isolates may be more difficult to eradicate in case of infection than HC isolates. Finally, we have been able to isolate *Trichosporon asteroides* and *Saccharomyces cerevisiae* only from RTT subjects. These isolates were both resistant to fluconazole (with MIC>64 μg/ml and MIC = 8 μg/ml respectively) while *Trichosporon asteroides* was also resistant to itraconazole (MIC>8 μg/ml). Such species are recognized as potential new emerging fungal pathogens [40] thus representing a potential threat for RTT subjects.

**Table 1:** Antifungals resistance of *Candida* isolates from HC or RTT patients

Species	Antifungals	Healthy controls (HC)				Rett syndrome (RTT) subjects			
		MIC (μg/ml)		^a^CBPs		MIC (μg/ml)		^a^CBPs	
		MIC_50_	MIC_90_	%S	%R	MIC_50_	MIC_90_	%S	%R
*C. albicans*	Fluconazole	0.5	> 64	75.6	24.4	1	2	75	25
	Itraconazole	0.25	> 8	36.6	63.4	0.0156	0.0156	100	0
	5-Flucytosine	0.125	0.5	97.6	2.4	0.125	0.125	100	0
*C. parapsilosis*	Fluconazole	0.5	2	92.9	7.1	2	> 64	64.3	35.7
	Itraconazole	0.0156	0.125	100	0	0.0156	> 8	64.3	35.7
	5-Flucytosine	0.125	0.5	100	0	0.125	0.125	100	0
^b^ *Candida spp.*	Fluconazole	0.125	0.25	100	0	> 64	> 64	0	100
	Itraconazole	0.0156	0.0156	100	0	8	8	0	100
	5-Flucytosine	0.125	0.125	100	0	0.125	0.125	100	0

^a^According to EUCAST recommendations; *S* sensible; *R* Resistant; MIC ranges: Fluconazole 0.125–64 μg/ml; Itraconazole 0.0156–8 μg/ml; 5-Flucytosine 0.125–64 μg/ml. ^b^*Candida* spp. isolated from RTT subjects (i.e. *C. glabrata, C. pararugosa* and *C. tropicalis*); *Candida* spp. isolated from HC (i.e. *C. deformans, C. intermedia* and *C. lusitaniae*)

### *C. parapsilosis* isolates from RTT subjects and HC are genetically distinct

**Fig. 2:**
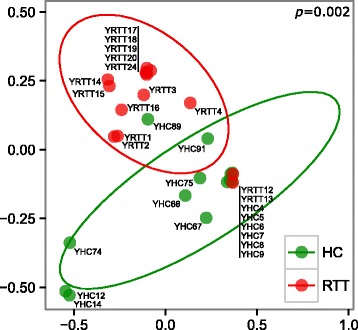
Multidimensional scaling analysis of *C. parapsilosis* genetic diversity calculated by UPGMA hierarchical clustering analysis of samples’ distance similarities (Jaccard index) from RAPD genotyping. *C. parapsilosis* isolates from HC and RTT subjects in green and red, respectively

The genetic diversity among *Candida* isolates was determined by UPGMA hierarchical clustering analysis of Jaccard distances calculated from RAPD genotyping. We observed that *C. parapsilosis* isolates from RTT samples were genetically unrelated to those from HC, with most of RTT *C. parapsilosis* isolates clustering in a single group (Fig. 2 and Additional file 4: Figure S3; *p* = 0.002, PERMANOVA). On the contrary, *C. albicans* isolates from RTT subjects were genetically more diverse, clustering in different clades of the tree (Additional file 5: Figure S4; *p* = 0.779, PERMANOVA). It is worth noting that we only obtained 4 *C. albicans* isolates from RTT samples.

### *Candida parapsilosis* from RTT subjects induces high levels of IL-10 in PBMCs

**Fig. 3:**
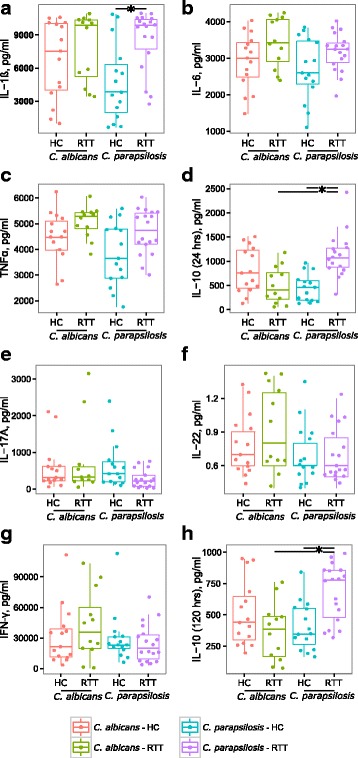
Cytokines production by peripheral blood mononuclear cells (PBMCs; 5 × 10^5^ cell) after stimulation with 5 × 10^6^ heat-killed *C. albicans* or *C. parapsilosis* isolates from HC and RTT subjects. In panels **a**-**d**) are reported the values for the cytokines produced by innate immune cells (IL-1β, IL-6, TNFα and IL-10 after 24 h of PBMCs stimulation) while in **e**-**h**) the values for the cytokines produced following adaptive immune responses (IL-17A, IL-22, IFNγ and IL-10 after 5 days of PBMCs stimulation). The dots represent each of the three replicates *per* isolate tested; *p < 0.05, Wilcoxon rank-sum test

The first step in the immunological response against *Candida* is the production by innate immune cells of pro-inflammatory cytokines, such as IL-1β, IL-6 and TNFα. Successively, these cytokines promote adaptive immunity mediated by Th1 or Th17 responses [2]. Stimulation of PBMCs with *C. albicans* and *C. parapsilosis* isolates from HC and RTT subjects revealed that RTT *Candida parapsilosis* isolates induced higher levels of IL-1β (*p* = 0.01, Wilcoxon rank-sum test) and, although only close to statistical significance, TNFα (*p* = 0.058, Wilcoxon rank-sum test) with respect to HC *Candida parapsilosis* isolates (Fig. 3). On the contrary, no significant differences were observed in the levels of IL-17A, IL-22 and INFγ (Fig. 3). Furthermore we observed that *C. parapsilosis* isolates from RTT subjects induced highly significant levels of IL-10 compared to HC *C. parapsilosis* isolates and RTT *C. albicans* isolates (*p* < 0.003, Wilcoxon rank-sum test; Fig. 3) suggesting an increased fungal tolerance towards these *C. parapsilosis* isolates, potentially favouring fungal persistence within the host. Nevertheless, we did not observe significant differences in the expression of IDO1 in PBMCs stimulated by *C. parapsilosis* isolates (median HC = 30%, IQR = 22.8–39.3%; median RTT = 44%, IQR = 29.1–48.3%; *p* = 0.24, Wilcoxon rank-sum test). Since we observed variable levels of Th-driving cytokines, we asked whether *Candida* isolates were able to induce a different Th1/Th17 polarization. Therefore, we measured the intracellular levels of the key transcription factors T-bet and RORγt (Additional file 6: Figure S5), involved in the differentiation of CD4^+^ naïve cells in Th1 and Th17 cell, respectively [41]. As previously observed in culture supernatants, we measured variable, but not statistically significant levels of T-bet and RORγt in response to the different *C. parapsilosis* and *C. albicans* isolates (Additional file 6: Figure S5), reflecting the potential of different strains to elicit an immune reaction at different extents. This could be due to the diverse immune reactivity shown by the different isolates of the same species, as previously reported [42, 43]. However, *C. parapsilosis* isolates from RTT subjects induced more CD4^+^ cells co-expressing both RORγt and T-bet compared to HC *C. parapsilosis* isolates (raw *p-value* = 0.04, FDR-corrected *p-value* = 0.12; Wilcoxon rank-sum test; Additional file 6: Figure S5c).

## Discussion

In the present study we show that in RTT, a multisystemic neurological disorder, faecal *C. parapsilosis* isolates hold phenotypic traits potentially favouring the previously observed low-grade intestinal inflammatory status [26]. Species-level analysis of the cultivable gut mycobiota revealed *C. parapsilosis* as the most abundant yeast species in RTT subjects, genetically distinct from HC *C. parapsilosis* isolates. Interestingly, RTT *C. parapsilosis* isolates were characterized by high levels of resistance to azoles antifungals. Furthermore, the high levels of IL-10 produced by PBMCs in response to RTT *C. parapsilosis* isolates suggest that these isolates have developed the capacity to persist within the host. IL-10 usually exert a homeostatic control to keep inflammation under control, although high levels of IL-10 are characteristic of chronic fungal infections dominated by non-resolving inflammation [2]. It has been observed that *C. albicans* induces host’s immunosuppression by increased IL-10 production by immune cells representing an important mechanisms in *Candida* pathogenesis [44]. Likewise, RTT *C. parapsilosis* isolates could escape immune clearance through a mechanism mediated by high levels of IL-10 that, in turn, could impair antifungal Th1 immunity, thus favouring a persistent intestinal colonization.

*C. parapsilosis* has been described as one of the leading causes of invasive candidiasis [45], being responsible of macrophage activation and allergic airways inflammation [46] and to be one of the dominant *Candida* species leading to dysbiosis in IBDs [47]. Fungal opportunistic infections are generally ascribed to defective host immunity, although they could require specific microbial population dysbiosis [48], as recently observed in RTT [26]. Recent studies indicated that fungal infections may originate from individual’s own commensal strains and that the ability of a commensal organism to produce disease is not merely a consequence of impaired host immunity [49]. Indeed, *C. albicans* passage through the GI tract results in a phenotypic switch in the so-called gastrointestinal induced transition cells, where virulence-associated genes are down-regulated enabling fungal adaptation for long-term survival in the large intestine [50]. Recent studies further show how strain specific differences in morphogenetic networks, regulating the switch from hyphal and yeast forms, subtend differences in their pathogenic potential, thus suggesting the importance to move the metagenomics analyses to the strain level [51]. Therefore, *C. parapsilosis* isolates from RTT subjects may be commensals potentially hazardous for the host due not only to RTT’s altered immunological status [19] but also by the presence of a dysbiotic gut microbiota [26]. Moreover, RTT *C. parapsilosis* isolates induced a higher proportion of a mixed Th1/Th17 cells population compared to HC *C. parapsilosis* isolates. Although Th1 and Th17 responses usually counter-regulate each other, there are increasing evidences of co-operation and dependency between these two immunological responses [52] which are involved in chronic, pro-inflammatory responses as observed in IBDs [53] potentially resulting in adaptive immunity against the commensal microbiota [54]. Interestingly, it has been previously shown that MeCP2 could actually play a regulatory role in T-cell resilience to inflammation [20]. Emerging evidence indicates that MeCP2 deficiency is able to lead to cytokine dysregulation including macrophage-related cytokines in *Mecp2*-null mice and RTT females [18, 19], although the understanding of the molecular mechanisms underlying this pro-inflammatory status remains elusive.

We have previously evidenced an inflammatory process in both RTT patients [19, 23] and animal models [55]. Biochemical analyses showed significant changes in the expression of acute phase response (APR) and immune system proteins in the serum of RTT subjects [23]. A direct relationship between MeCP2 and the immune system has been demonstrated, since MeCP2 is important for the differentiation of naïve CD4^+^ T cells into Th17 cells and for the commitment of naïve CD4^+^ T cells to the Th1 lineage [24]. Indeed, MeCP2 plays a critical role in promoting multiple cytokine-mediated signalling pathways through the MeCP2-miR-124-SOCS5 axis. This signalling pathway is required for the activation of signal transducer and activator of transcription 3 (STAT3) and STAT1 in CD4^+^ T cells, with consequent generation of Th17 cells [24]. The mechanisms underlying the inflammatory process appear to be related to a severe cytokine dysregulation, possibly reflecting a macrophage dysregulation/dysfunction, as previously suggested for *Mecp2*^−/−^ mice [56]. Furthermore, there is a critical interplay between inflammation and oxidative stress in the underlying mechanisms [57]. More recently, MeCP2 has been reported to act as an epigenetic regulator of immune and inflammatory responses during zebrafish development [58]. In this context, the bacterial and fungal microbiota dysbiosis demonstrated in our previous metataxonomics study from the same cohort of subjects [26] and the presence of putative virulent, pro-inflammatory intestinal *C. parapsilosis* strains could represent an additional factor in RTT’s gastrointestinal pathophysiology and subclinical inflammation.

## Conclusions

The gut mycobiota is emerging as a key player in maintaining the overall microbial community structure of the human gut, and main actor in host physiopathology [59]. Previous observations from our team indicated the presence of a subclinical inflammatory status [23], cytokine dysregulation [19] and intestinal dysbiosis [26] contributing to gastrointestinal symptoms in Rett syndrome. In the present manuscript, we moved our analysis to the strain level, investigating whether fungal isolates from RTT subjects may favour the sub-inflammatory status triggered by MeCP2 deficiency. Our results discovered *C. parapsilosis,* but not *C. albicans,* as the most abundant and potentially pro-inflammatory yeast species in the gut of RTT subjects. We propose that intestinal dysbiosis and the presence of pro-inflammatory *C. parapsilosis* strains could have a role in RTT’s gastrointestinal abnormalities laying the basis for the design of novel therapeutic strategies that, by targeting specific components of the gut microbiota, would restore eubiosis and intestinal physiology in RTT.

## Additional files

Additional file 1: **Table S1.** Phenotypic characteristics and antifungals susceptibility of fungal isolates. ^§^, MIC ranges: fluconazole 0.125–64 μg/ml; itraconazole 0.0156–8 μg/ml; 5-flucytosine 0.125–64 μg/ml; ^#^, 0 = non-invasive; 1 = poor invasive; 2 = invasive; 3 = very invasive. * measured by optical density at 570 nm; NA, not applicable. (DOCX 39 kb)

Additional file 2: **Figure S1.** Relative abundances of *Candida albicans* and *Candida parapsilosis* isolates in Rett syndrome subjects (RTT) and healthy controls (HC). The total abundance of all the other fungal isolates is also reported as “other species” (PDF 22 kb)

Additional file 3: **Figure S2. a**) Intestinal fungal isolates ability (or not) to produce hyphae or pseudo-hyphae in relationship with their ability to be invasive on YPD solid medium; **b**) biofilm production by intestinal fungal isolates from HC and RTT subjects; ***p < 0.0001, Wilcoxon rank-sum test. (PDF 35 kb)

Additional file 4: **Figure S3.** UPGMA hierarchical clustering of *C. parapsilosis* genetic diversity calculated by using samples’ distance similarities (Jaccard index) from RAPD genotyping. *C. parapsilosis* isolates from HC and RTT subjects in green and red, respectively. (PDF 15 kb)

Additional file 5: **Figure S4.** UPGMA hierarchical clustering of *C. albicans* genetic diversity calculated by using samples’ distance similarities (Jaccard index) from RAPD genotyping. *C. albicans* isolates from HC and RTT subjects in green and red, respectively; in gray the lab strain SC5314. (PDF 16 kb)

Additional file 6: **Figure S5.** Percentage of positive T-cells to T-bet, RORγt and both transcription factors T-bet and RORγt, as measured by intracellular staining and flow cytometry of PBMCs stimulated with **a, b, c**) *C. parapsilosis* isolates and **d, e, f**) *C. albicans* isolates from HC and RTT subjects. Cells were gated for CD4^+^ and data are given as percentage of total gated CD4^+^ cells. (PDF 41 kb)

## Abbreviations

CBP: Clinical breakpoint
HC: Healthy controls
IBDs: Inflammatory bowel diseases
IDO1: Indoleamine 2,3-dioxygenase 1
IL: Interleukin
INF: Interferon, IQR, interquartile range
MeCP2: Methyl-CpG binding protein 2
MIC: Minimum inhibitory concentration
PBMC: Peripheral blood mononuclear cells
RAPD: Random amplification of polymorphic DNA
RTT: Rett syndrome
Th: T-helper
TNF: Tumor necrosis factor
T-reg cell: Regulatory T cell
UPGMA: Unweighted pair group method with arithmetic mean
