# Supplementary material for: Intestinal Candida parapsilosis isolates from Rett syndrome subjects bear potential virulent traits and capacity to persist within the host
Source: BMC Gastroenterol. 2018 May 2;18:57. doi: 10.1186/s12876-018-0785-z (PMC5930502; doi:10.1186/s12876-018-0785-z)
Supplement: Supplementary file 1 — Table S1. Phenotypic characteristics and antifungals susceptibility of fungal isolates. §, MIC ranges: fluconazole 0.125–64 μg/ml; itraconazole 0.0156–8 μg/ml; 5-flucytosine 0.125–64 μg/ml; #, 0 = non-invasive; 1 = poor invasive; 2 = invasive; 3 = very invasive. * measured by optical density at 570 nm; NA, not applicable. (DOCX 39 kb) [file 12876_2018_785_MOESM1_ESM.docx]

**Additional file 1: Table S1:** Phenotypic characteristics and antifungals susceptibility of fungal isolates.

| **Subject** | **Status** | **isolate ID** | **Fluconazole^§^** | **Itraconazole^§^** | **5-flucytosine^§^** | **Biofilm*** | **Agar invasion^#^** | **Hyphae formation** | **Species** |
| --- | --- | --- | --- | --- | --- | --- | --- | --- | --- |
| HC21 | HC | YHC1 | 0.5 | 1 | 0.125 | 0.82 | 2 | no hyphae | *Torulaspora delbrueckii* |
| HC22 | HC | YHC2†ǂ | 1 | 2 | 0.125 | 0.66 | 2 | no hyphae | *Candida albicans* |
| HC22 | HC | YHC3 | 0.5 | 0.125 | 0.125 | 1.18 | 2 | Hyphae | *Candida albicans* |
| HC29 | HC | YHC4 | 0.5 | 0.0156 | 0.125 | 2.04 | 3 | Pseudohyphae | *Candida parapsilosis* |
| HC29 | HC | YHC5 | 0.125 | 0.0156 | 0.125 | 1.99 | 3 | Pseudohyphae | *Candida parapsilosis* |
| HC29 | HC | YHC6 | 0.5 | 0.0156 | 0.125 | 1.59 | 3 | Pseudohyphae | *Candida parapsilosis* |
| HC29 | HC | YHC7†ǂ | 0.5 | 0.0156 | 0.125 | 1.57 | 3 | Pseudohyphae | *Candida parapsilosis* |
| HC29 | HC | YHC8 | 0.5 | 0.0156 | 0.125 | 1.96 | 3 | Pseudohyphae | *Candida parapsilosis* |
| HC29 | HC | YHC9 | 0.5 | 0.0156 | 0.25 | 2.03 | 3 | Pseudohyphae | *Candida parapsilosis* |
| HC29 | HC | YHC10 | 0.5 | 1 | 0.125 | 1.44 | 3 | Pseudohyphae | *Rhodotorula mucilaginosa* |
| HC29 | HC | YHC11 | 0.25 | 0.0156 | 0.125 | 1.97 | 3 | Pseudohyphae | *Rhodotorula mucilaginosa* |
| HC31 | HC | YHC12† | 2 | 0.0156 | 1 | 0.93 | 3 | Pseudohyphae | *Candida parapsilosis* |
| HC31 | HC | YHC13 | NA | NA | NA | NA | NA | NA | *Candida albicans* |
| HC31 | HC | YHC14 | 0.5 | 0.0156 | 4 | 0.94 | 3 | Pseudohyphae | *Candida parapsilosis* |
| HC31 | HC | YHC15 | NA | NA | NA | NA | NA | NA | *Candida albicans* |
| HC31 | HC | YHC16 | 0.25 | 0.0156 | 64 | 1.09 | 0 | no hyphae | *Candida albicans* |
| HC31 | HC | YHC17 | NA | NA | NA | NA | 0 | no hyphae | *Candida albicans* |
| HC31 | HC | YHC18 | 1 | 0.031 | 0.125 | NA | 0 | no hyphae | *Rhodotorula mucilaginosa* |
| HC31 | HC | YHC19 | 0.25 | 0.0156 | 0.5 | 1.63 | 0 | no hyphae | *Candida lusitaniae* |
| HC31 | HC | YHC20 | 0.5 | 0.0156 | 0.5 | NA | 0 | no hyphae | *Rhodotorula mucilaginosa* |
| HC31 | HC | YHC21 | 0.5 | 0.0156 | 0.125 | 1.24 | 0 | no hyphae | *Rhodosporidium kratochvilovae* |
| HC31 | HC | YHC22 | 0.125 | 0.0156 | 0.125 | 1.57 | 0 | no hyphae | *Candida albicans* |
| HC31 | HC | YHC23 | NA | NA | NA | NA | NA | NA | *Candida albicans* |
| HC31 | HC | YHC24 | NA | NA | NA | NA | NA | NA | *Candida albicans* |
| HC31 | HC | YHC25 | NA | NA | NA | NA | NA | NA | *Candida albicans* |
| HC31 | HC | YHC26 | 0.25 | 0.0156 | 0.5 | 0.20 | 0 | no hyphae | *Candida lusitaniae* |
| HC31 | HC | YHC27 | 0.125 | 0.0156 | 0.125 | 0.23 | 1 | no hyphae | *Candida albicans* |
| HC31 | HC | YHC28 | NA | NA | NA | 0.34 | 1 | no hyphae | *Candida albicans* |
| HC31 | HC | YHC29 | NA | NA | NA | NA | 3 | Pseudohyphae | *Candida parapsilosis* |
| HC31 | HC | YHC30 | NA | NA | NA | NA | 1 | no hyphae | *Candida albicans* |
| HC32 | HC | YHC31 | 0.125 | 0.0156 | 0.5 | 0.25 | 0 | no hyphae | *Pichia manshurica* |
| HC32 | HC | YHC32 | 0.25 | 0.0156 | > 64 | 0.47 | 2 | no hyphae | *Pichia manshurica* |
| HC32 | HC | YHC33 | NA | NA | NA | NA | 2 | Hyphae | *Pichia manshurica* |
| HC32 | HC | YHC34 | 0.25 | 0.0156 | 8 | 0.62 | 0 | no hyphae | *Pichia manshurica* |
| HC32 | HC | YHC35 | > 64 | > 8 | 0.125 | 0.54 | 0 | no hyphae | *Pichia manshurica* |
| HC32 | HC | YHC36 | NA | NA | NA | NA | NA | NA | *Pichia manshurica* |
| HC32 | HC | YHC37 | 0.5 | 0.0156 | 0.5 | 0.30 | 2 | no hyphae | *Pichia manshurica* |
| HC32 | HC | YHC38 | 0.25 | 0.0156 | 0.125 | 0.30 | 1 | no hyphae | *Pichia manshurica* |
| HC32 | HC | YHC39 | 0.25 | 0.0156 | 0.125 | 0.67 | 2 | Pseudohyphae | *Pichia manshurica* |
| HC32 | HC | YHC40 | > 64 | > 8 | 0.125 | 0.18 | 1 | no hyphae | *Pichia manshurica* |
| HC32 | HC | YHC41 | NA | NA | NA | NA | 2 | no hyphae | *Pichia manshurica* |
| HC33 | HC | YHC42 | NA | NA | NA | NA | 0 | no hyphae | *Rhodotorula mucilaginosa* |
| HC33 | HC | YHC43 | > 64 | > 8 | 0.125 | 0.24 | 1 | no hyphae | *Candida albicans* |
| HC33 | HC | YHC44 | > 64 | > 8 | 0.125 | NA | 3 | Hyphae | *Candida albicans* |
| HC33 | HC | YHC45 | 0.25 | 0.0156 | 0.125 | 0.14 | 3 | Hyphae | *Candida albicans* |
| HC33 | HC | YHC46 | 0.5 | 0.0156 | 0.125 | NA | 3 | Hyphae | *Candida albicans* |
| HC33 | HC | YHC47 | 0.25 | 0.0156 | 0.125 | 0.24 | 2 | no hyphae | *Pichia manshurica* |
| HC33 | HC | YHC48 | > 64 | > 8 | 0.125 | 0.57 | 1 | no hyphae | *Candida albicans* |
| HC33 | HC | YHC49 | 0.25 | 0.0156 | 0.125 | 0.61 | 3 | no hyphae | *Candida albicans* |
| HC33 | HC | YHC50 | 0.25 | 0.0156 | 0.125 | 0.26 | 3 | Hyphae | *Candida albicans* |
| HC33 | HC | YHC51 | 0.25 | 0.0156 | 0.125 | 0.41 | 3 | Hyphae | *Candida albicans* |
| HC33 | HC | YHC52 | > 64 | > 8 | 0.125 | 0.17 | 0 | Hyphae | *Rhodosporidium kratochvilovae* |
| HC34 | HC | YHC53 | > 64 | > 8 | 0.125 | 0.18 | 3 | no hyphae | *Candida albicans* |
| HC37 | HC | YHC54 | 0.25 | 1 | 0.5 | 0.41 | 3 | Hyphae | *Candida albicans* |
| HC37 | HC | YHC55 | 0.25 | 2 | 0.5 | 0.71 | 1 | Hyphae | *Candida albicans* |
| HC37 | HC | YHC56†ǂ | 0.25 | 2 | 0.5 | 0.42 | 1 | Hyphae | *Candida albicans* |
| HC37 | HC | YHC57 | 0.5 | 2 | 0.5 | 0.75 | 1 | Hyphae | *Torulaspora delbrueckii* |
| HC37 | HC | YHC58 | 0.5 | 2 | 0.5 | 0.35 | 2 | Hyphae | *Candida albicans* |
| HC38 | HC | YHC59 | 0.125 | 0.0156 | 0.125 | 0.30 | 1 | no hyphae | *Candida deformans* |
| HC39 | HC | YHC60† | > 64 | > 8 | 0.125 | 0.39 | 0 | no hyphae | *Candida albicans* |
| HC39 | HC | YHC61 | > 64 | 0.125 | 0.125 | 0.16 | 3 | Hyphae | *Candida albicans* |
| HC39 | HC | YHC62 | > 64 | > 8 | 0.125 | 0.38 | 0 | no hyphae | *Candida albicans* |
| HC39 | HC | YHC63 | > 64 | 0.125 | 0.125 | 0.19 | 3 | Hyphae | *Candida albicans* |
| HC39 | HC | YHC64 | > 64 | > 8 | 0.125 | 0.36 | 0 | no hyphae | *Candida albicans* |
| HC39 | HC | YHC65 | 32 | 1 | 0.125 | 0.33 | 1 | no hyphae | *Candida albicans* |
| HC41 | HC | YHC66† | 0.25 | 0.0156 | 0.125 | 0.31 | 2 | Pseudohyphae | *Candida parapsilosis* |
| HC41 | HC | YHC67 | 1 | 0.0156 | 0.125 | 0.90 | 2 | Pseudohyphae | *Candida parapsilosis* |
| HC41 | HC | YHC68 | > 64 | 0.0156 | 0.125 | NA | 3 | Hyphae | *Aspergillus glaucus* |
| HC44 | HC | YHC69 | 0.125 | 0.125 | 0.125 | 0.27 | 1 | Hyphae | *Candida albicans* |
| HC44 | HC | YHC70 | 0.125 | 0.0156 | 0.125 | 0.13 | 2 | no hyphae | *Candida albicans* |
| HC44 | HC | YHC71 | 0.125 | 0.25 | 0.125 | 0.20 | 0 | Hyphae | *Torulaspora delbrueckii* |
| HC44 | HC | YHC72 | 0.5 | 0.25 | 0.125 | 0.18 | 0 | no hyphae | *Candida albicans* |
| HC44 | HC | YHC73 | 0.5 | 0.25 | 0.125 | 0.21 | 2 | Hyphae | *Candida albicans* |
| HC44 | HC | YHC74 | 0.125 | 0.0156 | 0.125 | 0.63 | 3 | Pseudohyphae | *Candida parapsilosis* |
| HC44 | HC | YHC75†ǂ | > 64 | 0.125 | 0.5 | 0.17 | 2 | no hyphae | *Candida parapsilosis* |
| HC46 | HC | YHC76 | 0.5 | 2 | 0.125 | 0.40 | 3 | Hyphae | *Candida albicans* |
| HC46 | HC | YHC77 | 0.5 | 2 | 0.125 | 0.76 | 2 | Hyphae | *Candida albicans* |
| HC46 | HC | YHC78 | 0.5 | 2 | 0.125 | 1.42 | 2 | Hyphae | *Candida albicans* |
| HC46 | HC | YHC79† | 0.5 | 2 | 0.125 | 0.29 | 2 | Hyphae | *Candida albicans* |
| HC47 | HC | YHC80 | 0.125 | 0.0156 | 0.125 | 0.20 | 2 | Hyphae | *Torulaspora delbrueckii* |
| HC47 | HC | YHC81 | 0.25 | 1 | 0.125 | 0.29 | 2 | Hyphae | *Candida albicans* |
| HC47 | HC | YHC82 | 0.25 | 0.125 | 0.125 | 0.55 | 2 | Hyphae | *Torulaspora delbrueckii* |
| HC47 | HC | YHC83 | 0.25 | 1 | 0.125 | 0.19 | 3 | Hyphae | *Candida albicans* |
| HC50 | HC | YHC84 | 0.125 | 0.0156 | 0.125 | 2.54 | 2 | no hyphae | *Candida intermedia* |
| HC50 | HC | YHC85 | 0.125 | 0.0156 | 0.125 | 0.69 | 1 | no hyphae | *Candida lusitaniae* |
| HC50 | HC | YHC86 | 0.5 | 0.0156 | 0.125 | 0.86 | 1 | Hyphae | *Candida albicans* |
| HC50 | HC | YHC87 | 0.25 | 0.0156 | 0.125 | 0.44 | 1 | Pseudohyphae | *Rhodotorula mucilaginosa* |
| HC50 | HC | YHC88 | 0.25 | 0.0156 | 0.125 | 1.05 | 1 | no hyphae | *Candida lusitaniae* |
| HC50 | HC | YHC89 | 0.5 | 0.125 | 0.125 | 0.67 | 1 | no hyphae | *Candida parapsilosis* |
| HC50 | HC | YHC90 | 0.5 | 0.125 | 0.125 | 0.56 | 1 | no hyphae | *Candida lusitaniae* |
| HC50 | HC | YHC91† | 0.5 | 0.0156 | 0.125 | 0.65 | 3 | Pseudohyphae | *Candida parapsilosis* |
| HC50 | HC | YHC92 | NA | NA | NA | NA | NA | NA | *Yarrowia lipolytica* |
| HC52 | HC | YHC93 | 0.25 | 0.0156 | 0.125 | 0.63 | 1 | Hyphae | *Candida albicans* |
| HC52 | HC | YHC94 | 0.25 | 0.0156 | 0.125 | 0.70 | 1 | Hyphae | *Candida albicans* |
| HC52 | HC | YHC95 | 0.125 | 0.0156 | 0.125 | 0.90 | 1 | Hyphae | *Candida albicans* |
| HC52 | HC | YHC96† | 0.5 | 0.0156 | 0.125 | 0.54 | 3 | Hyphae | *Candida albicans* |
| HC53 | HC | YHC97 | 0.5 | 0.0156 | 0.125 | 1.07 | 2 | Hyphae | *Candida albicans* |
| HC53 | HC | YHC98 | 0.5 | 2 | 0.125 | 0.81 | 1 | Hyphae | *Candida albicans* |
| RTT9 | RTT | YRTT1 | > 64 | 2 | 0.125 | 2.42 | 2 | Pseudohyphae | *Candida parapsilosis* |
| RTT9 | RTT | YRTT2 | > 64 | 2 | 0.125 | 2.20 | 2 | Pseudohyphae | *Candida parapsilosis* |
| RTT9 | RTT | YRTT3 | 64 | > 8 | 0.125 | 0.60 | 2 | Pseudohyphae | *Candida parapsilosis* |
| RTT9 | RTT | YRTT4†ǂ | 1 | 0.0625 | 0.125 | 1.49 | 2 | no hyphae | *Candida parapsilosis* |
| RTT9 | RTT | YRTT5†ǂ | 1 | 0.0156 | 0.125 | 0.81 | 0 | no hyphae | *Candida albicans* |
| RTT9 | RTT | YRTT6† | 1 | 0.0156 | 0.125 | 0.35 | 0 | no hyphae | *Candida albicans* |
| RTT10 | RTT | YRTT7†ǂ | 4 | 0.0156 | 0.125 | 0.77 | 1 | no hyphae | *Candida albicans* |
| RTT35 | RTT | YRTT8 | > 64 | 2 | 0.125 | 2.35 | 2 | Hyphae | *Candida tropicalis* |
| RTT35 | RTT | YRTT9 | 8 | 0.0156 | 0.125 | 1.33 | 3 | Hyphae | *Saccharomyces cerevisiae* |
| RTT35 | RTT | YRTT10 | > 64 | 8 | 0.125 | 1.79 | 3 | Hyphae | *Candida tropicalis* |
| RTT73 | RTT | YRTT11†ǂ | 2 | 0.0156 | 0.125 | 1.77 | 2 | no hyphae | *Candida albicans* |
| RTT73 | RTT | YRTT12† | 2 | 0.0156 | 0.125 | 2.04 | 2 | no hyphae | *Candida parapsilosis* |
| RTT73 | RTT | YRTT13 | 2 | 0.0156 | 0.125 | 2.10 | 2 | no hyphae | *Candida parapsilosis* |
| RTT116 | RTT | YRTT14† | 16 | > 8 | 0.125 | 1.04 | 1 | no hyphae | *Candida parapsilosis* |
| RTT147 | RTT | YRTT15 | 1 | 0.0156 | 0.125 | 0.68 | 2 | Pseudohyphae | *Candida parapsilosis* |
| RTT147 | RTT | YRTT16†ǂ | 2 | 0.0156 | 0.125 | 0.93 | 2 | no hyphae | *Candida parapsilosis* |
| RTT147 | RTT | YRTT17 | 1 | 0.0156 | 0.125 | 1.46 | 2 | Pseudohyphae | *Candida parapsilosis* |
| RTT147 | RTT | YRTT18 | 1 | 0.0156 | 0.125 | 0.97 | 2 | Pseudohyphae | *Candida parapsilosis* |
| RTT147 | RTT | YRTT19 | 1 | 0.0156 | 0.125 | 0.82 | 2 | Pseudohyphae | *Candida parapsilosis* |
| RTT163 | RTT | YRTT20† | > 64 | > 8 | 0.125 | 0.91 | 2 | no hyphae | *Candida parapsilosis* |
| RTT199 | RTT | YRTT21 | > 64 | > 8 | 0.125 | 0.94 | 3 | Hyphae | *Trichosporon asteroides* |
| RTT199 | RTT | YRTT22 | > 64 | > 8 | 0.125 | 0.74 | 2 | no hyphae | *Candida pararugosa* |
| RTT199 | RTT | YRTT23 | > 64 | > 8 | 0.125 | 0.83 | 0 | no hyphae | *Candida glabrata* |
| RTT199 | RTT | YRTT24†ǂ | 0.125 | 0.0156 | 0.125 | 1.88 | 0 | Pseudohyphae | *Candida parapsilosis* |

†, isolates used for cytokine assays; ǂ, isolates analysed by flow cytometry; ^§^, MIC ranges: fluconazole, 0.125-64 μg/ml; itraconazole, 0.0156-8 μg/ml; 5-flucytosine, 0.125-64 μg/ml; ^#^, 0= non-invasive; 1= poor invasive; 2= invasive; 3= very invasive. *, measured by optical density at 570nm; NA, not applicable.
