# Supplementary figures and images for: Intestinal Candida parapsilosis isolates from Rett syndrome subjects bear potential virulent traits and capacity to persist within the host
Source: BMC Gastroenterol. 2018 May 2;18:57. doi: 10.1186/s12876-018-0785-z (PMC5930502; doi:10.1186/s12876-018-0785-z)

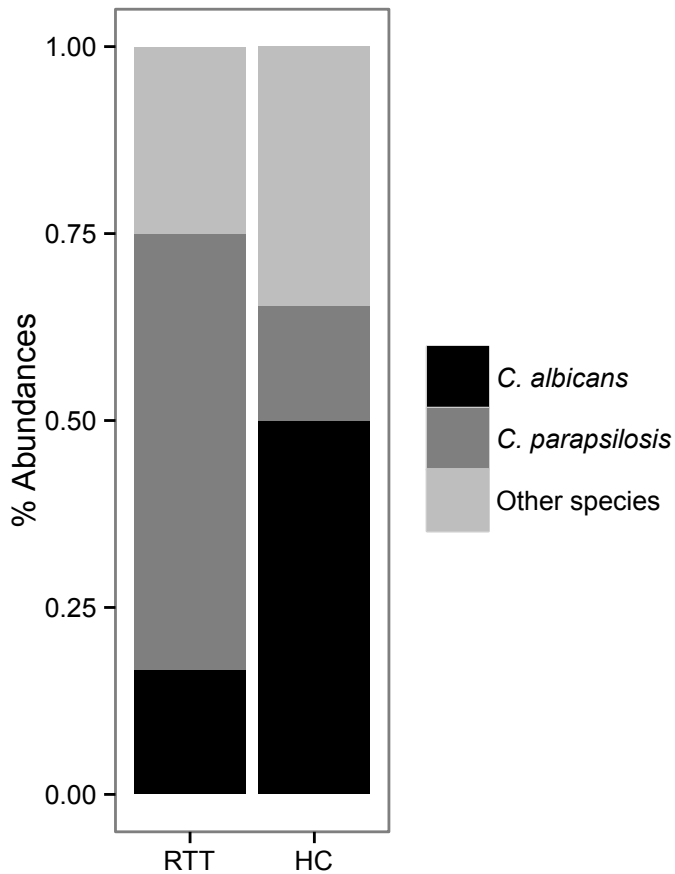

Supplement: Supplementary file 2 — Figure S1. Relative abundances of Candida albicans and Candida parapsilosis isolates in Rett syndrome subjects (RTT) and healthy controls (HC). The total abundance of all the other fungal isolates is also reported as “other species” (PDF 22 kb) [file 12876_2018_785_MOESM2_ESM.pdf]

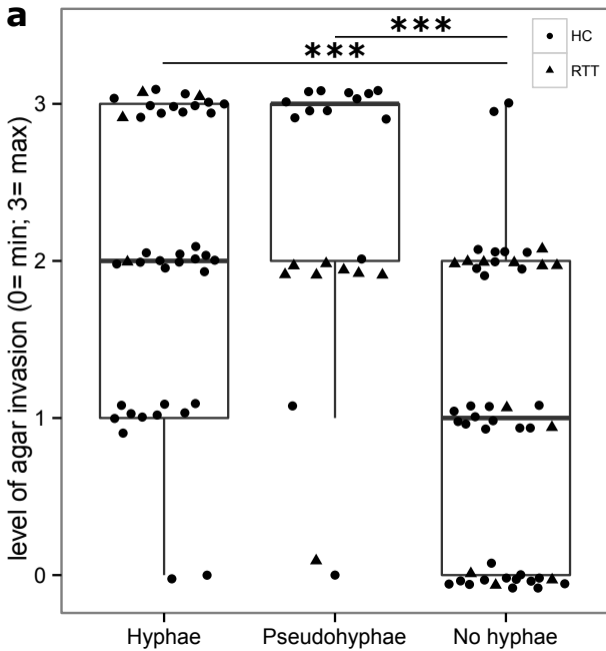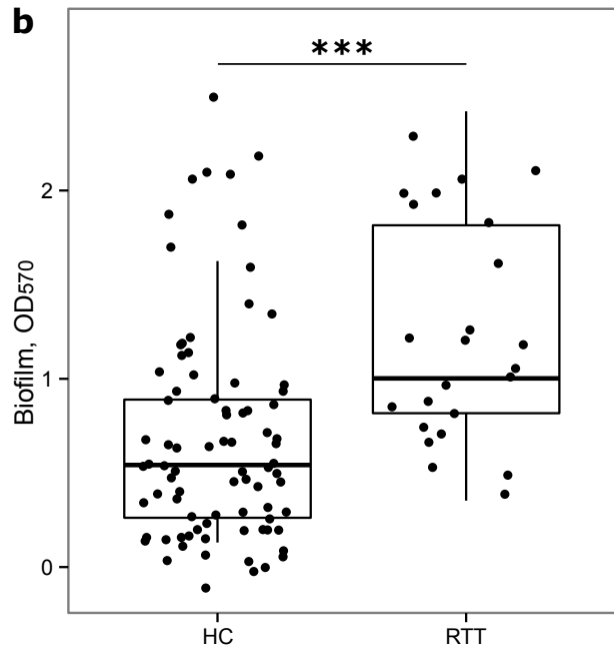

Supplement: Supplementary file 3 — Figure S2. a) Intestinal fungal isolates ability (or not) to produce hyphae or pseudo-hyphae in relationship with their ability to be invasive on YPD solid medium; b) biofilm production by intestinal fungal isolates from HC and RTT subjects; ***p < 0.0001, Wilcoxon rank-sum test. (PDF 35 kb) [file 12876_2018_785_MOESM3_ESM.pdf]

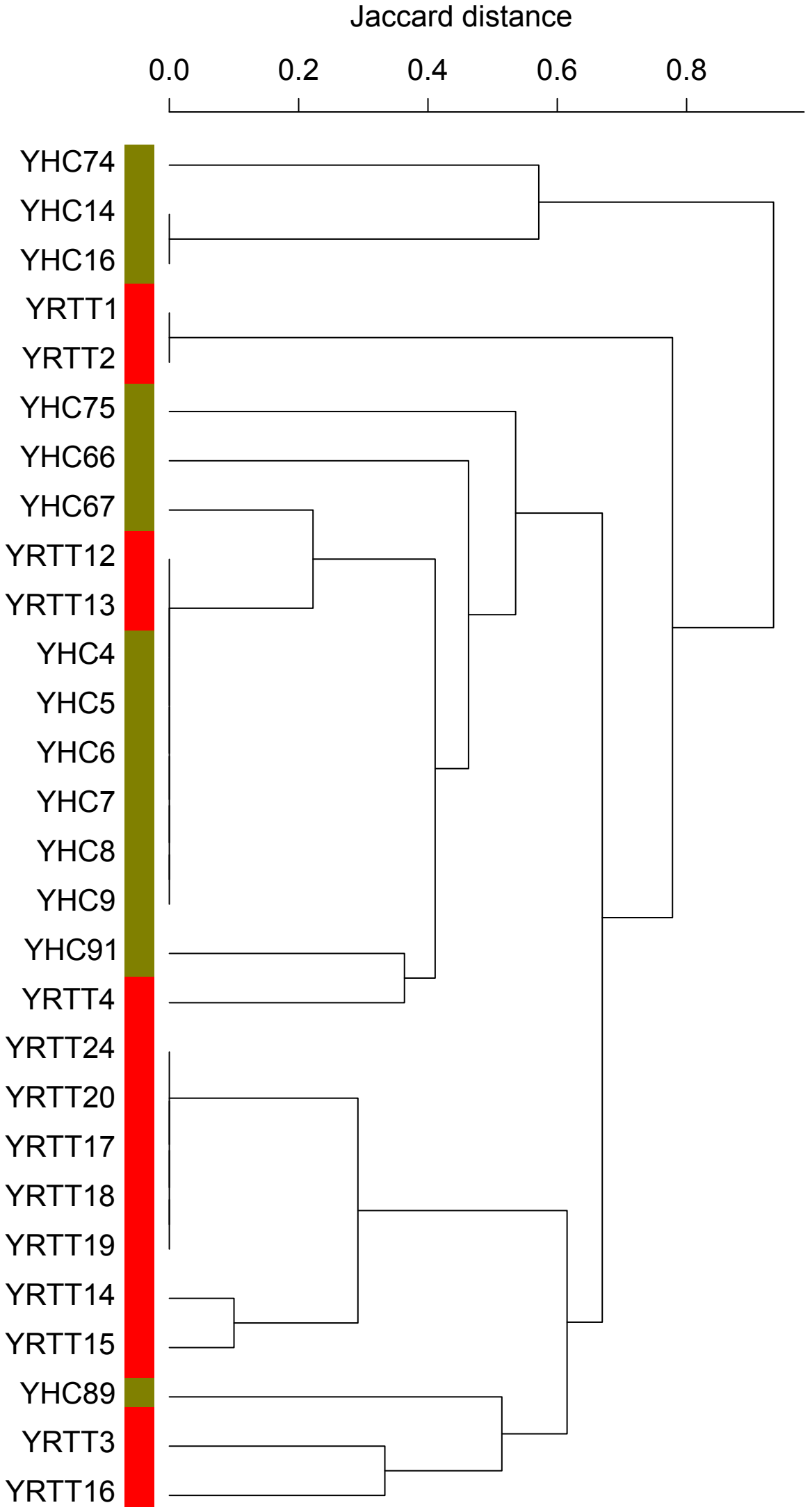

Supplement: Supplementary file 4 — Figure S3. UPGMA hierarchical clustering of C. parapsilosis genetic diversity calculated by using samples’ distance similarities (Jaccard index) from RAPD genotyping. C. parapsilosis isolates from HC and RTT subjects in green and red, respectively. (PDF 15 kb) [file 12876_2018_785_MOESM4_ESM.pdf]

## Jaccard distance

0.0

0.2

0.4

0.6

0.8

1.0

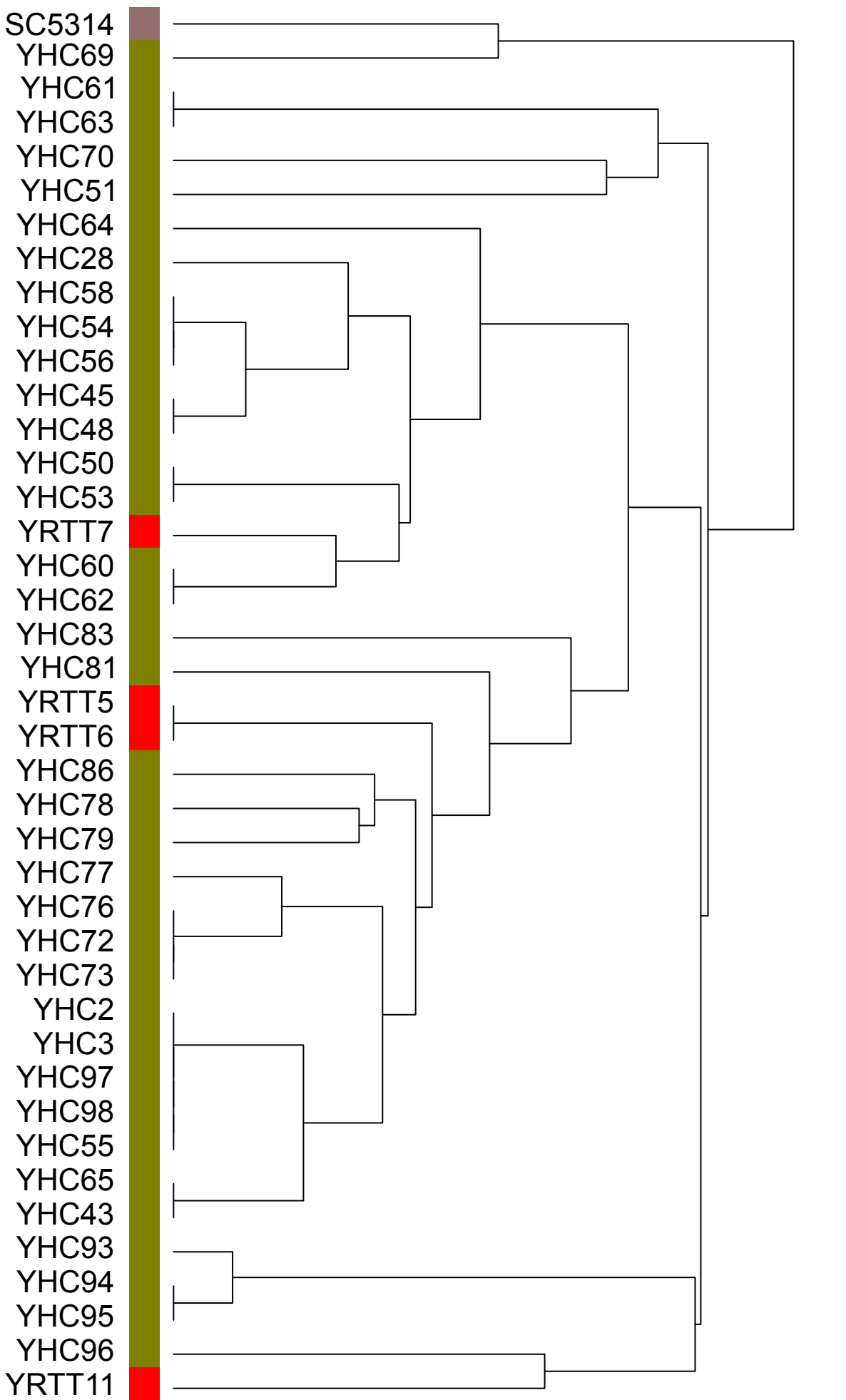

Supplement: Supplementary file 5 — Figure S4. UPGMA hierarchical clustering of C. albicans genetic diversity calculated by using samples’ distance similarities (Jaccard index) from RAPD genotyping. C. albicans isolates from HC and RTT subjects in green and red, respectively; in gray the lab strain SC5314. (PDF 16 kb) [file 12876_2018_785_MOESM5_ESM.pdf]

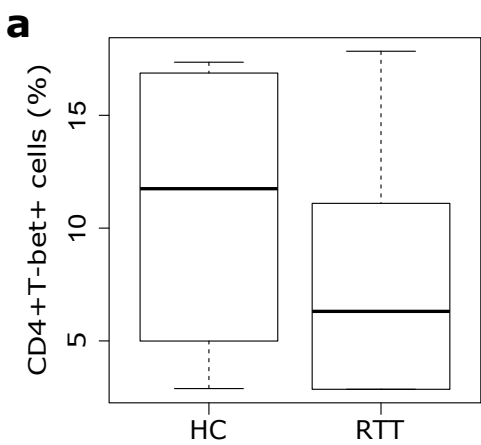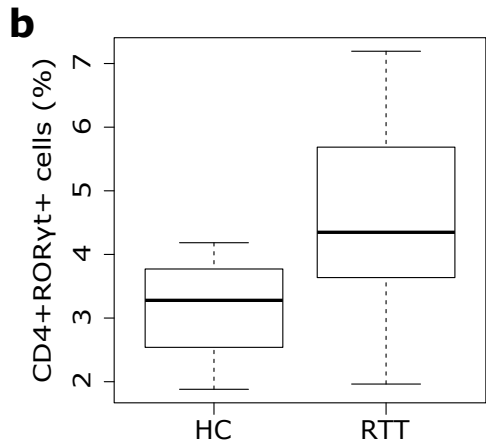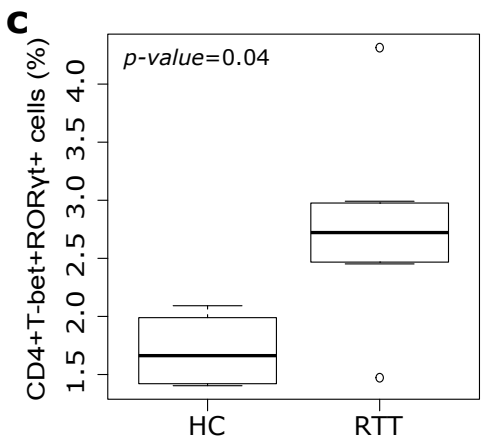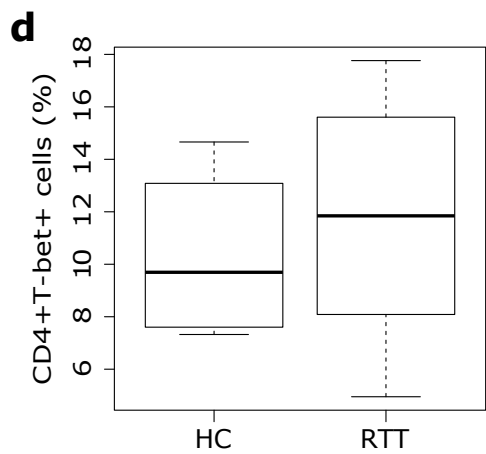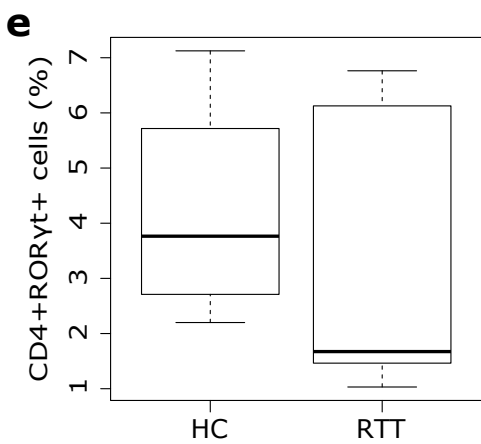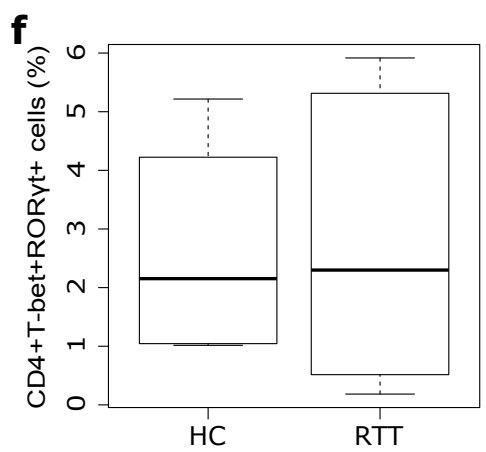

Supplement: Supplementary file 6 — Figure S5. Percentage of positive T-cells to T-bet, RORγt and both transcription factors T-bet and RORγt, as measured by intracellular staining and flow cytometry of PBMCs stimulated with a, b, c) C. parapsilosis isolates and d, e, f) C. albicans isolates from HC and RTT subjects. Cells were gated for CD4+ and data are given as percentage of total gated CD4+ cells. (PDF 41 kb) [file 12876_2018_785_MOESM6_ESM.pdf]
